# The knowledge, attitudes and practice of nasal irrigation among patients with rhinosinusitis: a cross-sectional study

**DOI:** 10.3389/falgy.2025.1741401

**Published:** 2026-02-06

**Authors:** Feng-ling Yang, Biao Wang, Wei Deng, Zhen-hua Jiang, Li-jun Zhang, Ni Liao, Lun-shu Shen

**Affiliations:** 1 Department of Otorhinolaryngology-Head and Neck Surgery, Mianyang Central Hospital, School of Medicine, University of Electronic Science and Technology of China, Mianyang, Sichuan, China; 2 Department of Ophthalmology, The Third Hospital of Mianyang, Sichuan Mental Health Center, Mianyang, Sichuan, China

**Keywords:** knowledge, attitudes and practice, nasal irrigation, patient education, rhinosinusitis

## Abstract

**Objective:**

Nasal inflammatory diseases significantly impair patients' quality of life, with global prevalence varying regionally. Nasal irrigation, endorsed by international guidelines as adjunctive therapy, lacks standardized protocols and patient education, potentially compromising efficacy. This study evaluated the knowledge, attitudes, and practice regarding nasal irrigation in patients with rhinosinusitis and identified factors influencing adherence, with the objective of informing evidence-based strategies to improve patient education and clinical management.

**Methods:**

A cross-sectional survey was conducted at a tertiary hospital via a 40-item questionnaire assessing the knowledge, attitudes, practice and information sources related to nasal irrigation among 233 patients with nasal inflammatory diseases.

**Results:**

The participants exhibited significant knowledge gaps in solvent/solute selection, concentration, temperature, irrigation devices, shelf life, and clinical indications of nasal irrigation (correct answer rate <60%). Younger participants (<50 years) demonstrated a better understanding of temperature, frequency, device differences and pediatric applicability. The attitudes were favorable: 88.7% perceived nasal irrigation as safe, and 92.6% acknowledged its importance; however, only 58.4% believed it could independently treat rhinosinusitis. Practice rates were high (80.4%), with 94.1% performing self-administered irrigation. Hospitals were the primary information source (75.5%), whereas younger, educated patients more frequently utilized online platforms and science/professional literature.

**Conclusion:**

Despite high adherence and positive perceptions, critical knowledge gaps persist in solution parameters, device use, and clinical applications. Age- and education-stratified communication, which integrate multimedia resources and hospital-led guidance, are essential for addressing disparities and enhancing treatment efficacy, particularly among older, less educated and read populations.

## Introduction

Nasal inflammatory diseases encompass a spectrum of conditions, including acute and chronic rhinitis, acute and chronic sinusitis, and specific subtypes such as allergic rhinitis and drug-induced rhinitis. Epidemiological data suggest that the prevalence of these conditions varies significantly, with rhinitis affecting 10% to 40% and sinusitis affecting 5% to 15% of the population across different regions (1, 2). These diseases are frequently associated with symptoms such as nasal obstruction, rhinorrhea, and cephalalgia, which profoundly impact patients' quality of life.

Nasal irrigation is a critical adjunctive therapy for nasal inflammatory diseases. Both the EPOS2020 and Rhinitis 2020 guidelines recommend its use in the management of various forms of rhinitis and sinusitis in adults and children (1, 2). Similarly, the 2018 Chinese guideline for chronic sinusitis endorses nasal saline irrigation as an adjunctive treatment for chronic and refractory sinusitis across all age groups (3). As a safe and feasible intervention, nasal irrigation is widely utilized both pre- and post-operatively in endoscopic sinus surgery. It functions by eliminating nasal and sinus secretions, crusts, antigens, biofilms, and inflammatory mediators, thereby enhancing mucociliary clearance and significantly alleviating nasal symptoms. These effects collectively contribute to improved patient quality of life (4, 5).

Despite extensive guidelines and consensuses underscoring the importance of nasal irrigation in managing nasal inflammatory diseases, healthcare professionals often regard it as a simplistic intervention, delegating its execution entirely to patients. Currently, there is a lack of standardized nursing protocols for nasal irrigation, and minimal effort is devoted to educating patients about its principles, procedural techniques, and precautions. As a result, it remains uncertain whether patients perform nasal irrigation correctly, possess confidence in the treatment, or implement it effectively. To date, few studies have explored the knowledge, attitudes, and practice of patients with sinonasal inflammatory diseases regarding nasal irrigation, despite the close relationship between these factors and the therapeutic efficacy of the intervention (6). The evidence suggests that when patients receive adequate education about nasal irrigation, their attitudes toward the treatment improve, leading to more thorough implementation and, consequently, better clinical outcomes (7).

Currently, there is no consensus regarding key parameters of nasal irrigation, including solute, solvent, volume, pressure, frequency, posture, and device selection. Nevertheless, several studies have reviewed and summarized the existing evidence on nasal irrigation techniques (4, 8). Building on these reviews, this study aims to develop a self-designed questionnaire to assess the knowledge, attitudes, and practice of nasal irrigation among patients with nasal inflammatory diseases. By evaluating the current implementation of nasal irrigation in this patient population, the study seeks to optimize patient education and clinical management, ultimately enhancing therapeutic outcomes for nasal inflammatory diseases.

## Methods

This study is a cross-sectional investigation involving patients diagnosed with nasal inflammatory diseases, including acute(˂12 weeks) and chronic(≥12 weeks) rhinosinusitis(inflammation of the nose and/or paranasal sinuses) (1). Participants were recruited from the Department of Otolaryngology at Mianyang Central Hospital between March 15, 2025 and May 31, 2025. During their hospital stay or follow-up visits post-discharge, patients completed an anonymous, paper-based questionnaire. The study was reviewed and approved by the Ethics Committee of Mianyang Central Hospital (No. S20250337-01). All procedures in this study were conducted in accordance with the ethical principles outlined in the Declaration of Helsinki. Prior to participation, written informed consent was obtained from all participants.

### Setting and population

Patients with nasal inflammatory diseases were provided with a paper-based questionnaire by nursing staff during their hospital stay or follow-up visits post-discharge. The participants were required to complete the questionnaire independently. The inclusion criteria of the present study are as follows: 1. Patients diagnosed with nasal inflammatory diseases, including acute or chronic rhinosinusitis. 2. Patients with experience in using nasal irrigation (either currently or in the past). Exclusion Criteria include: 1. Illiterate individuals unable to complete the questionnaire. 2. Patients who could not adequately comprehend the questionnaire content, defined as the ability to answer fewer than 50% of the questions.

### Sampling methodology

Prior to the formal initiation of this study, a pilot study was conducted involving approximately 30 patients (responses from these participants were excluded from the final analysis). The pilot study primarily evaluated the accuracy rate of responses to questions assessing knowledge of nasal irrigation, which was determined to be 50%. The sample size for the main study was calculated via the following formula: $n=(Z_{a/2}^{2}*p*(1-p))/E2$, where a 95% confidence interval was selected ($Z_{a/2}=1.96$); p=0.50 (estimated accuracy rate); and *E* (margin of error), which was set at 0.10. On the basis of this calculation, a minimum sample size of 97 was needed. Considering potential nonresponses or incomplete questionnaires and subsequent multifactorial analysis, the study plans to increase the sample size by 200–250.

### Questionnaire design

As no established or validated questionnaires are available, a self-designed questionnaire was developed for this study on the basis of a review of the relevant literature and expert interviews. The questionnaire, detailed in Supplementary Appendix S1, comprises five sections with a total of 40 items: (1) general information (6 items, including age, sex, occupation, education level, reading frequency, and reading preferences), (2) knowledge of nasal irrigation (18 items), (3) attitudes toward nasal irrigation (4 items), (4) practice of nasal irrigation (8 items), and (5) sources of nasal irrigation information and equipment (4 items). The knowledge section was designed with reference to review articles by Jin et al. and Succar et al. (4, 8). Prior to finalization, the questionnaire was reviewed and discussed by a small team of otolaryngology and nursing professionals to ensure its relevance, clarity and validity.

### Statistical analysis

The results are statistically presented as frequencies and medians with interquartile ranges (IQRs). Data analysis was performed via SPSS 21.0 (SPSS Inc., Chicago, USA). For categorical data, comparisons were made via the chi-square test or Fisher's exact test, as appropriate. For continuous data with nonnormal distributions, the Mann–Whitney *U* test was used for comparisons between two groups, and the Kolmogorov–Smirnov test was applied for comparisons among multiple groups. Multivariate analyses were conducted via negative binomial regression or logistic regression models. A *P* value of less than 0.05 was considered statistically significant.

## Results

### Descriptive statistics

After 21 questionnaires with missing response rates exceeding 50% were excluded, a total of 233 questionnaires were included in the final analysis. The age of the participants ranged from 13 to 86 years, with 104 (44.6%) being female. The occupational distribution was as follows: farmers (66, 28.7%), freelancers (60, 26.1%), professionals (42, 18.3%), and workers (28, 12.2%). With respect to educational attainment, the majority of the participants had completed middle school (104, 45.4%) or held a college degree or higher (89, 38.9%). In terms of reading frequency, 134 patients (60.4%) read occasionally, whereas 55 (24.8%) reported no reading habits. The most common types of reading materials included news (67, 25.4%) and stories or fictions (60, 22.7%), with fewer patients reading professional (40, 15.2%) or popular scientific (26, 9.8%) books. The detailed results are presented in Table 1.

**Table 1:** Demographic characteristics of the participants.

Variables	*n*(%)/M(IQR)
Participants involved	233
Age (years)	48 (34, 57)
Sex	
Male	129 (55.4)
Female	104 (44.6)
Occupation	
Farmer	66 (28.7)
Worker	28 (12.2)
Freelancer	60 (26.1)
Professionals	42 (18.3)
Civil servant	10 (4.3)
Student	10 (4.3)
Without jobs	14 (6.1)
Educational attainment	
Elementary school or below	36 (15.7)
Middle school	104 (45.4)
College or above	89 (38.9)
Reading frequency	
Frequently	33 (14.9)
Occasionally	134 (60.3)
None	55 (24.8)
Types of reading	
Story or fiction	60 (22.7)
News	67 (25.4)
Popular scientific readings	26 (9.8)
Professional readings	40 (15.2)
Others	71 (26.9)

M, median; IQR, interquartile ranges.

### Knowledge of nasal irrigation

**Figure 1:**
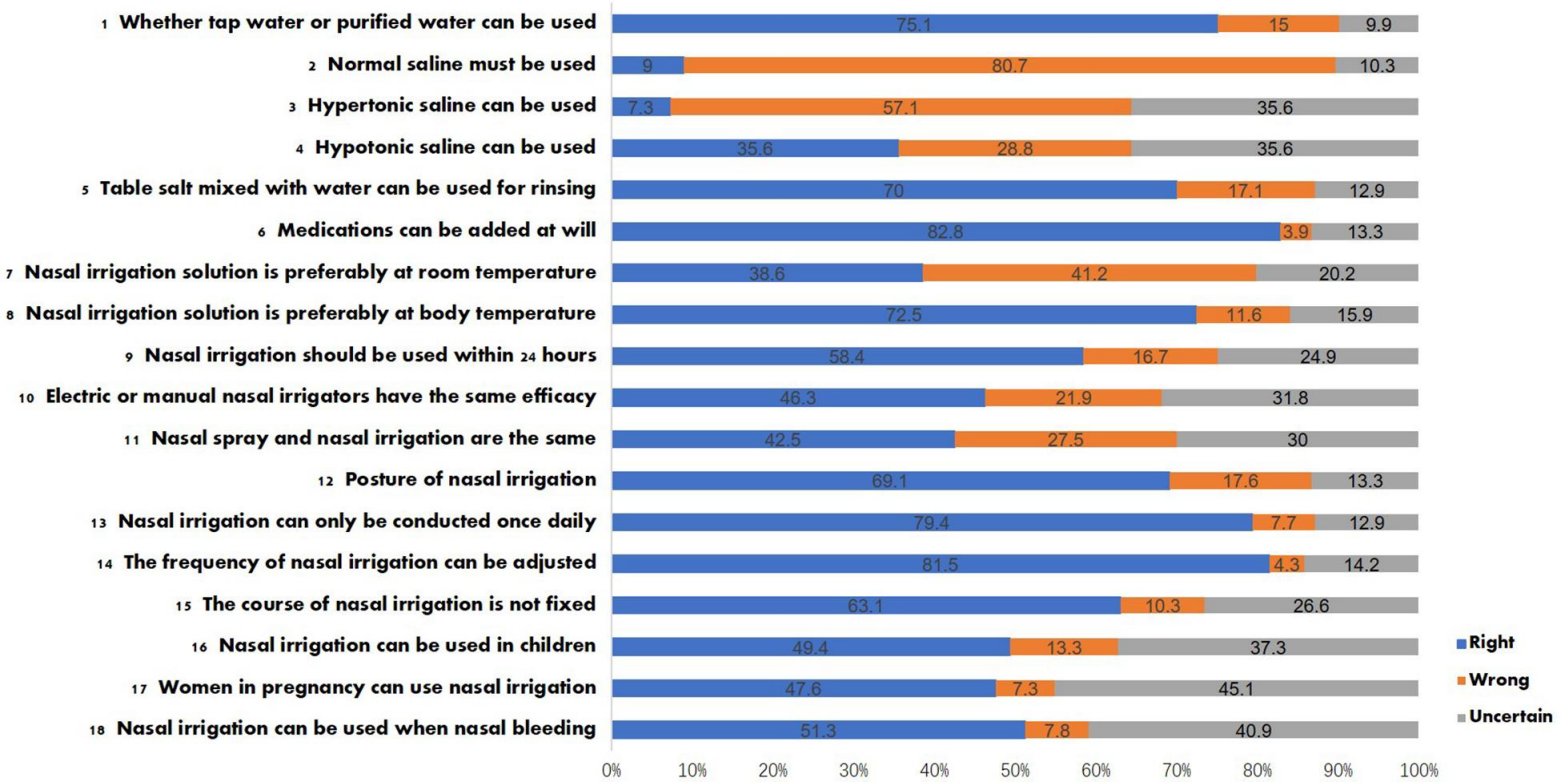
Answers of the knowledge about nasal irrigation.

This study primarily assessed patients' knowledge of nasal irrigation, encompassing aspects such as solvent and solute selection, concentration, temperature, shelf time, irrigation devices, posture, frequency, treatment duration, indications, and target populations. Items with a correct response rate exceeding 80% included “Medications can be added at will” (Item 6, 193, 82.8%) and “The frequency of nasal irrigation can be adjusted” (Item 14, 190, 81.5%). In contrast, the items with a correct response rate below 60% were as follows: “Normal saline must be used” (Item 2, 21, 9.0%), “Hypertonic saline can be used” (Item 3, 17, 7.3%), “Hypotonic saline can be used” (Item 4, 83, 35.6%), “Nasal irrigation solution is preferably at room temperature” (Item 7, 90, 38.6%), “Nasal irrigation solution should be used within 24 h” (Item 9, 136, 58.4%), “Electric and manual nasal irrigators have the same efficacy” (Item 10, 108, 46.3%), “Nasal spray and nasal irrigation are the same” (Item 11, 99, 42.5%), “Nasal irrigation can be used in children” (Item 16, 115, 49.4%), “Women in pregnancy can use nasal irrigation” (Item 17, 111, 47.6%), and “Nasal irrigation can be used when nasal bleeding” (Item 18, 119, 51.3%). The results of the knowledge assessment are illustrated in Figure 1.

### Attitudes toward nasal irrigation

In the survey assessing attitudes toward nasal irrigation, 205 patients (88.7%) perceived it as safe (including responses of “definitely safe” and “safe”), whereas only 3 patients (1.3%) considered it unsafe. The majority of participants (211, 92.6%) regarded nasal irrigation as important (including “very important” and “important”) for the management of rhinosinusitis. Furthermore, 184 patients (79.0%) reported that nasal irrigation could aid in their recovery from rhinosinusitis. However, only 136 patients (58.4%) believed that nasal irrigation could be used to treat rhinosinusitis. The detailed results regarding attitudes toward nasal irrigation are presented in Table 2.

**Table 2:** Attitudes toward nasal irrigation.

Items	Attitudes [*n*(%)]				
Item 1	Definitely safe	Safe	Neutral	Unsafe	Definitely unsafe
Nasal irrigation is safe					
	98 (42.4)	107 (46.3)	23 (10.0)	3 (1.3)	0
Item 2	Yes	No	Uncertain		
Nasal irrigation can treat rhinosinusitis	136 (58.4)	49 (21.0)	48 (20.6)		
Item 3	Very important	Important	Neutral	Unimportant	Definitely
Unimportant					
Nasal irrigation is important for rhinosinusitis treatment					
	129 (56.6)	82 (36.0)	14 (6.1)	1 (0.4)	2 (0.9)
Item 4	Yes	No	Uncertain		
Nasal irrigation can help you relieve from rhinosinusitis	184 (79.0)	19 (8.1)	30(12.9)		

### Nasal irrigation practice

Among the surveyed patients, 124 (60.8%) reported having used nasal irrigation, and 164 patients (80.4%) were currently using it. The majority of patients (191, 94.1%) were able to perform nasal irrigation independently, and 187 (92.6%) believed that it alleviated their symptoms. Although 56 patients (28.6%) experienced discomfort during nasal irrigation, 191 (94.6%) reported being able to tolerate it. When faced with discomfort, 16 patients (7.8%) chose to continue irrigation, 126 (61.8%) opted to delay it, and 58 (28.4%) decided to stop. Additionally, 152 patients (74.5%) expressed a willingness to recommend nasal irrigation to others experiencing nasal discomfort. The detailed results regarding the practice of nasal irrigation are presented in Table 3.

**Table 3:** Practice of nasal irrigation.

Items	Practice [*n*(%)]			
Item 1	Yes	No		
Have you ever used nasal irrigation?	124 (60.8)	80 (39.2)		
Item 2	Yes	No		
Are you using nasal irrigation now?	164 (80.4)	40 (19.6)		
Item 3	Yes	No		
Nasal irrigation can be conducted by yourself.	191 (94.1)	12 (5.9)		
Item 4	Yes	No	Uncertain	
Nasal irrigation can alleviate your discomfort.	187 (92.6)	2 (1.0)	13 (6.4)	
Item 5	Yes	No		
Nasal irrigation makes you uncomfortable.	56 (28.6)	140 (71.4)		
Item 6	Yes	No		
You can tolerate nasal irrigation.	191 (94.6)	11 (5.4)		
Item 7	Stop irrigation	Continue irrigation	Delay irrigation	Uncertain
What would you do if nasal irrigation made you uncomfortable?				
	58 (28.4)	16 (7.8)	126 (61.8)	4 (2.0)
Item 8	Yes	No	Uncertain	
You would recommend nasal irrigation to others with nasal discomfort.	152 (74.5)	25 (12.3)	27(13.2)	

### Sources of nasal irrigation information and equipment

Among the surveyed patients, 182 (75.5%) reported learning about nasal irrigation from hospitals, and 178 (66.2%) acquired their knowledge of nasal irrigation through hospital-based sources. When encountering issues related to nasal irrigation, the majority of patients (241, 73.3%) indicated that they would seek guidance from medical staff. Additionally, most patients (133, 61.3%) reported purchasing nasal irrigation devices online. The detailed results regarding the sources of information and devices for nasal irrigation are presented in Table 4.

**Table 4:** Acquisition of the information and equipment about nasal irrigation.

Items	Sources of acquisition[*n*(%)]			
Item 1	Hospital	Internet	Paper-based literature	Acquaintances
Where did you know about nasal irrigation?				
	182 (75.5)	27 (11.2)	15 (6.2)	17 (7.1)
Item 2	Hospital	Internet	Paper-based literature	Acquaintances
Where did you learn about the knowledge of nasal irrigation?				
	178 (66.2)	42 (15.6)	36 (13.4)	13 (4.8)
Item 3	Hospital	Internet	Pharmacy	
Where did you buy your nasal irrigation device?	23 (10.6)	133 (61.3)	61 (28.1)	
Item 4	Hospital	Internet	Paper-based literature	People irrigated
If you have problems about nasal irrigation, whom do you like to seek help from?				
	241 (73.3)	30 (9.1)	34 (10.3)	24 (7.3)

### Factors affecting mastery in nasal irrigation knowledge

By employing negative binomial regression on the basis of the number of accurately answered questions, it was discerned that patient age, sex, educational attainment and reading frequency did not significantly influenced the proficiency of nasal irrigation knowledge, as delineated in Supplementary Table S1.

Multiple logistic regression analysis was subsequently employed to assess the impact of various factors on responses to individual items related to nasal irrigation knowledge. The analysis revealed that the correct response rates for the following items were significantly higher among patients under 50 years of age than among those aged 50 and above (*P* < 0.05): “Medications can be added at will”, “Nasal irrigation solution is preferably at body temperature”, “Electric or manual nasal irrigators have the same efficacy”, “Nasal spray and nasal irrigation are the same”, “Nasal irrigation can only be conducted once daily” and “Nasal irrigation can be used in children”. Additionally, the frequency of reading significantly influenced patients' understanding of “Nasal irrigation can only be conducted once daily” and “Nasal irrigation can be used when nasal bleeding”, with a higher reading frequency associated with increased correct response rates (*P* < 0.05). Sex and educational level did not significantly affect patients' mastery of nasal irrigation knowledge. For detailed results, refer to Supplementary Table S2.

### Factors influencing attitudes toward nasal irrigation

No statistically significant differences were observed in the influence of age, sex, educational level, or reading frequency on patients' attitudes toward nasal irrigation, as detailed in Supplementary Table S3.

### Factors affecting the practice of nasal irrigation

Multiple logistic regression analysis revealed that patient age significantly influenced the response to two questions: “Nasal irrigation can alleviate your discomfort” and “What would you do if nasal irrigation made you uncomfortable?”. Among patients under 50 years of age, 110 (85.3%) reported that nasal irrigation could relieve nasal discomfort, whereas 18 (14.0%) expressed uncertainty (including those who selected “uncertain” or provided no response). In contrast, among patients aged 50 years or older, 77 (74.8%) indicated that nasal irrigation alleviated nasal discomfort, and 25 (24.3%) were uncertain. A statistically significant difference in the perception of efficacy of nasal irrigation in improving nasal discomfort was observed between the two age groups (*P* = 0.018).

Among patients under 50 years of age, when nasal irrigation caused discomfort, 34 (26.4%) chose to discontinue irrigation, 5 (3.9%) opted to continue, 81 (62.8%) decided to delay irrigation, and 9 (7.0%) were uncertain (including those who selected “uncertain” or provided no response). In contrast, among patients aged 50 years or older, 24 (23.3%) chose to discontinue, 11 (10.7%) opted to continue, 45 (43.7%) decided to delay, and 23 (22.3%) were uncertain (including those who selected “uncertain” or provided no response). A statistically significant difference was observed in the responses of the two age groups when they experienced discomfort from nasal irrigation (*P* = 0.018). Sex, educational attainment, and reading frequency did not significantly influence patients' practice regarding nasal irrigation. The impacts of these factors on nasal irrigation practice are detailed in Table 5.

**Table 5:** Factors influencing the practice of nasal irrigation.

Items	Age		Sex		Educational level		Reading frequency	
	(<50 years/≥50 years)		(male/female)		(elementary school or below/ middle school/ college or above)		(frequently/occasionally/none)	
	*χ*²	*P*	χ²	*P*	χ²	*P*	χ²	*P*
Item 1	3.856	0.145	3.453	0.178	2.254	0.895	5.895	0.435
Have you ever used nasal irrigation?								
Item 2	2.060	0.217	3.152	0.207	8.141	0.228	4.906	0.556
Are you using nasal irrigation now?								
Item 3	3.026	0.220	1.616	0.446	9.032	0.172	11.575	0.072
Nasal irrigation can be conducted by yourself.								
Item 4	10.015	**0** **.** **018**	0.845	0.839	4.443	0.880	12.816	0.171
Nasal irrigation can alleviate your discomfort.								
Item 5	3.733	0.155	1.695	0.429	2.444	0.875	11.528	0.073
You can tolerate nasal irrigation.								
Item 6	1.131	0.568	0.173	0.917	5.862	0.439	8.868	0.439
Nasal irrigation makes you uncomfortable.								
Item 7	11.865	**0** **.** **018**	2.396	0.663	15.172	0.232	15.234	0.229
What would you do if nasal irrigation made you uncomfortable?								
Item 8	3.306	0.347	3.577	0.311	6.469	0.692	8.316	0.503
You would recommend nasal irrigation to others with nasal discomfort.								

Values in bold: *P* < 0.05.

### Factors affecting the acquisition of nasal irrigation

This study demonstrated a significant age-related disparity in knowledge acquisition about nasal irrigation, with patients under 50 years of age primarily obtaining information from hospitals (61.5%), the internet (18.3%), and paper-based literature (17.8%), whereas patients aged 50 years and above predominantly relied on hospitals (74.3%), with lower utilization of the internet (10.9%) and paper-based literature (5.9%) (*P* = 0.018).

Educational level significantly influences how patients with rhinosinusitis learn about nasal irrigation (“Where did you know about nasal irrigation?”), acquire related knowledge (“Where did you learn about the knowledge of nasal irrigation?”), and seek help with nasal irrigation-related questions (“If you have problems about nasal irrigation, whom do you like to seek help from?”). Statistically significant differences were observed in the acquisition ways across the three educational level groups (*P* < 0.05). The detailed results are presented in Table 6.

**Table 6:** Factors influencing acquisition of nasal irrigation.

Items	Age		Sex		Educational level		Reading frequency	
	(<50 years/≥50 years)		(male/female)		(elementary school or below/ middle school/ college or above)		(frequently/occasionally/none)	
	χ²	*P*	χ²	*P*	χ²	*P*	χ²	*P*
Item 1	1.905	0.592	3.571	0.312	25.422	**<0** **.** **001**	7.401	0.285
Where did you know about nasal irrigation?								
Item 2	10.076	**0** **.** **018**	3.685	0.298	18.393	**0** **.** **005**	10.616	0.101
Where did you learn about the knowledge of nasal irrigation?								
Item 3	4.080	0.130	1.146	0.564	1.012	0.908	1.774	0.777
Where did you buy your nasal irrigation device?								
Item 4	6.633	0.085	2.053	0.562	18.407	**0** **.** **005**	8.409	0.210
If you have problems about nasal irrigation, whom do you like to seek help from?								

Values in bold: *P* < 0.05.

**Figure 2:**
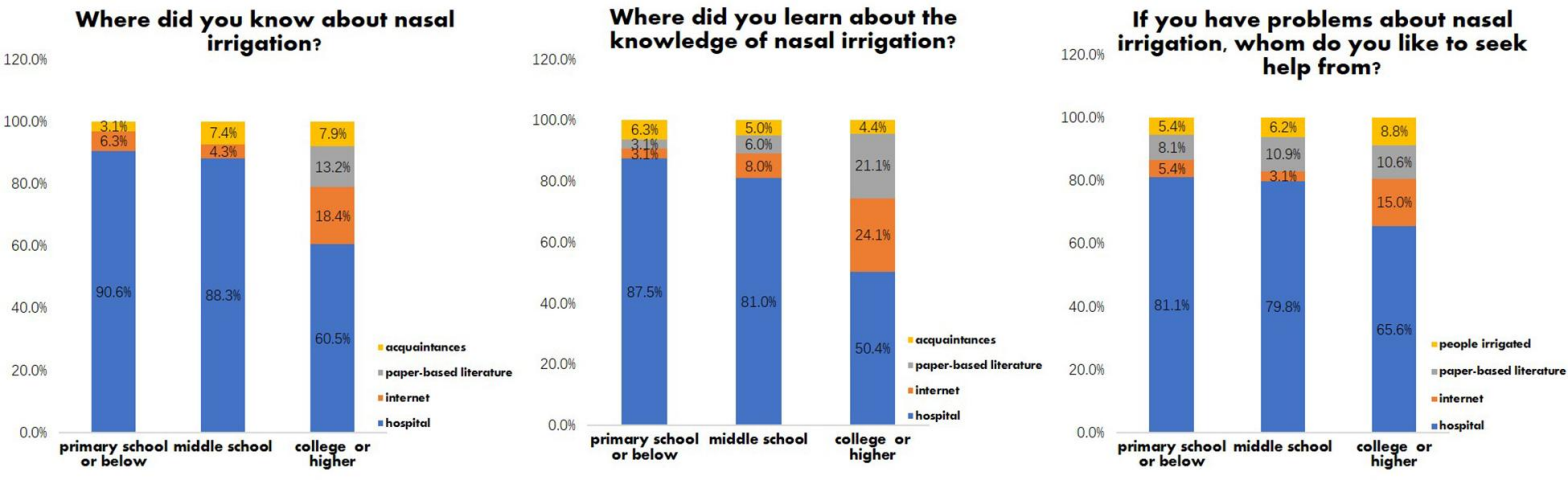
Educational level influencing the information acquisition of nasal irrigation.

Regarding sources for learning about nasal irrigation, patients with a primary school education or below relied predominantly on hospitals (90.6%), with minimal engagement with the internet (6.3%), acquaintances (3.1%) or literature (0%). Middle school-educated patients similarly prioritized hospitals (88.3%), whereas college-educated individuals demonstrated diversified sourcing, combining hospitals (60.5%) with the internet (18.4%), literature (13.2%) and acquaintances (7.9%). With respect to knowledge acquisition channels, hospital dependency remained highest among the primary school or below (87.5%) and middle school groups (81.0%), although the latter showed increased use of the internet (8.0%) and literature (6.0%). College-or-above-educated patients exhibited a tripartite pattern: hospitals (50.4%), the internet (24.1%), and literature (21.1%), with less reliance on acquaintances (4.4%). When encountering difficulties, hospital assistance dominated across all groups (81.1% in primary school or below, 79.8% in middle school, and 65.6% in the college or above group), yet college-or-above-educated patients reported greater utilization of the internet (15.0% vs. ≤5.4% in lower-educated groups), literature (10.6% vs. ≤8.1% in lower-educated groups), and a greater proportion of seeking help from irrigated people (8.8% vs. ≤6.2% in lower-educated groups). The data are shown in Figure 2. Sex and reading frequency did not significantly influence the ways in which patients acquired information about nasal irrigation.

## Discussion

This study employed a cross-sectional survey to analyze the knowledge, attitudes, and practice of nasal irrigation among patients with rhinosinusitis admitted to a tertiary hospital. This study aimed to explore the awareness and practice of nasal irrigation from the perspective of patients with rhinosinusitis, a topic that, to our knowledge, has not been previously investigated on the basis of a review of the literature.

With respect to knowledge about nasal irrigation, patients demonstrated a relatively good understanding of whether solutes can be freely added to the nasal irrigation solution and the recommended treatment duration. However, significant knowledge gaps have been identified in several areas, including the optimal concentration and temperature of the irrigation solution, the target populations and indications for nasal irrigation, the distinctions between manual and electric irrigation devices, and the differences between nasal irrigation and nasal sprays.

The optimal concentration of nasal irrigation solution remains a subject of debate, posing significant challenges for patients seeking effective management. Previous studies have employed saline solutions of varying concentrations, with some utilizing sodium lactate Ringer's solution. Both hypertonic and isotonic saline solutions are commonly used in clinical practice, as supported by the literature. While hypertonic saline may offer advantages in relieving symptom and enhancing ciliary motility, no significant differences have been observed in imaging outcomes or olfactory improvement. Consequently, the most effective concentration for nasal irrigation remains undetermined (9–12). Further complicating this issue is the availability of hypertonic solutions with varying concentrations on the market, raising questions about the need for a standardized hypertonic concentration (13). Patients, particularly elderly patients, often lack sufficient knowledge regarding the appropriate concentration of nasal irrigation solutions. This has led to the misconception that any saline solution is suitable for nasal irrigation. In clinical practice, we have observed instances where patients prepare homemade saline solutions using table salt, which may result in inconsistent concentrations and potentially compromise nasal function.

The temperature of the irrigation solution is a critical factor in ensuring patient comfort and safety. To minimize nasal mucosal irritation or bleeding, solutions close to body temperature are recommended (14). While some studies suggest that premade irrigation solutions can be refrigerated for up to 12 days without an increased risk of cross-contamination (15), evidence regarding the optimal expiration date for nasal irrigation solutions remains limited, and no consensus has been established. Given the potential for bacterial growth, it is advisable to use prepared nasal irrigation solutions promptly. Some studies recommend that solutions be used within 24 h to maintain hygiene and reduce the risk of contamination (9, 14).

Manual (e.g., squeeze bottles or bulb syringes) and electric (e.g., powered irrigation) devices, despite differing in their power mechanisms, are both classified as high-flow, high-pressure systems. These devices facilitate thorough nasal cavity cleansing, making them particularly suitable for patients with chronic sinusitis who present with significant nasal secretions and crusts (9, 16). Recent advancements in nasal irrigation devices have focused on enhancing irrigation efficacy, yet all of these advancements remain grounded in the high-flow, high-pressure principle (17). In contrast, nasal sprays operate as low-volume, high-pressure devices, primarily designed for daily nasal moisturization. They are less effective at removing nasal secretions, crusts, and inflammatory mediators and are incapable of achieving the deep cleansing and sinus penetration provided by nasal irrigation systems. Consequently, nasal sprays are fundamentally distinct from nasal irrigation devices in both function and clinical application (18). The distinctions between manual and electric irrigation devices, as well as between nasal irrigation and nasal sprays, are critical for patients when selecting appropriate nasal care products. For example, relying solely on nasal sprays for post sinusitis surgical care is insufficient.

The survey data in the present study underscore the need for enhanced patient education regarding nasal irrigation, particularly concerning the solution concentration, temperature, and shelf-life. Additionally, tailored guidance should be provided to patients on the basis of their specific disease conditions to ensure optimal adjuvant treatment outcomes. Nasal irrigation is a safe and versatile intervention suitable for a broad range of demographics, including infants, children, and pregnant women (19, 20). By communicating with patients about the safety and applicability of nasal irrigation across these populations, clinicians can bolster patient confidence, improve treatment compliance, and enhance therapeutic efficacy.

This study revealed that patient age and reading frequency significantly influence patients' understanding of nasal irrigation practice. Age has a broad impact, affecting knowledge across multiple domains, including solute composition, solution temperature, irrigation frequency, treatment duration, target populations, and distinctions between electric and manual irrigation devices, as well as between nasal irrigation and nasal sprays. Patients aged 50 years and above demonstrated significantly lower correct-answer rates on some topics, likely attributable to age-related declines in cognitive and comprehension abilities, as well as limited access to knowledge and information sources. Furthermore, patients with higher reading frequencies exhibited better comprehension of key aspects of nasal irrigation, such as the optimal irrigation frequency and appropriate clinical indications. These findings underscore the need for targeted educational interventions, particularly for elderly patients and those with less reading engagement. To enhance accessibility and engagement, educational materials should incorporate visual aids, such as illustrations or videos, and minimize text-heavy content, thereby making the information more intuitive and easier to understand.

This study revealed that patients exhibit a high level of confidence in the safety of nasal irrigation, with 88.7% recognizing it as a safe intervention. Additionally, the majority of patients (92.6%) acknowledged its importance in the management of rhinosinusitis, and 79.0% believed that it contributed to their recovery. However, only 58.4% expressed confidence in its efficacy as a standalone treatment for rhinosinusitis. This discrepancy may reflect patient perceptions that, while nasal irrigation is a valuable adjunct to rhinosinusitis treatment, it is insufficient as a sole therapeutic modality.

Nasal irrigation is widely adopted among patients with nasal inflammatory diseases, with a utilization rate of 80.4%. This high adoption rate is largely driven by recommendations from healthcare providers during hospitalization or follow-up visits. Furthermore, the majority of these patients in the present study have undergone surgical interventions, which contributes to their high compliance with nasal irrigation practice. Overall, nasal irrigation is considered easy to perform, as 94.1% of patients reported being able to complete the procedure independently. Although 28.6% of patients experienced discomfort during nasal irrigation, 94.6% found it tolerable. Notably, only 28.4% of patients discontinued irrigation after experiencing discomfort, whereas approximately two-thirds (69.6%) chose to continue or delay the procedure. These findings underscore the high safety and tolerability of nasal irrigation as a therapeutic intervention. Age-related differences were also observed. A greater proportion of patients under 50 years of age reported that nasal irrigation alleviated their discomfort. In contrast, patients aged 50 years and above were more likely to continue irrigation despite discomfort, potentially reflecting their greater resilience, which may be attributed to more challenging life experiences.

In this study, patients primarily obtained information about nasal irrigation from hospitals, with a relatively low proportion relying on the internet, literature or acquaintances. Similarly, hospitals were the main resource for resolving irrigation-related issues, highlighting the central role of healthcare providers in patient education regarding nasal irrigation in Southwest China. Evidence-based nursing programs have been shown to improve the efficacy of nasal irrigation in chronic sinusitis patients following functional endoscopic sinus surgery, leading to significant improvements in symptoms, CT scores, nasal endoscopy scores, and overall quality of life (21). These findings underscore the importance of providing patients with scientifically validated, evidence-based clinical guidance on nasal irrigation practice.

Younger patients and those with higher educational levels demonstrated greater independence in accessing information, often utilizing the internet or books rather than relying solely on hospitals. For this demographic, educational outreach should extend beyond hospital settings to include traditional media (e.g., books, newspapers, television, radio) and new media platforms (e.g., social media, self-published content) to broaden the reach of nasal irrigation knowledge. In contrast, older patients and those educated less may benefit more from concise, easily accessible educational materials available through hospital platforms, such as posters and instructional videos. Tailoring educational strategies to address the specific needs and preferences of different patient groups can enhance the effectiveness of nasal irrigation practice and improve clinical outcomes.

This study has several limitations. First, as a pilot study designed to identify key areas for subsequent clinical work and public education, the sample size was relatively small. Future studies should aim to include a more diverse population, encompassing individuals of varying ages and educational backgrounds. The current study included a limited representation of children and highly educated individuals, potentially omitting some critical issues. For pediatric populations, a more tailored questionnaire—such as one assessing children's resistance to nasal irrigation, which is completed by parents—could be developed to design more acceptable nasal irrigation protocols (22). Additionally, this study was conducted at a tertiary hospital in Southwest China. While tertiary hospitals serve a large and diverse patient population, regional disparities exist between Southwest China and coastal areas. Variations in educational levels and access to information between these regions could influence patient knowledge and practice related to nasal irrigation. Therefore, caution should be exercised when extrapolating these results to other settings. To address these limitations, future research should involve large-scale, multicenter surveys across diverse geographic regions. Furthermore, the differences between age groups observed in this study resulted from an exploratory subgroup analysis. The rationale for this dichotomization requires further validation in prospective studies. Such efforts would enhance the robustness and applicability of the findings, providing a more comprehensive understanding of nasal irrigation practice and their implications for patient care.

## Conclusion

This study evaluated the attitudes, knowledge, and practice of nasal irrigation among patients with nasal inflammatory diseases who were undergoing or had undergone inpatient treatment at a tertiary hospital. The findings revealed that patients held a highly positive attitude toward the safety and efficacy of nasal irrigation. The practice rate of nasal irrigation was high, with most patients able to perform the procedure independently. Hospitals and healthcare providers were the primary sources of information for patients regarding nasal irrigation.

However, patients demonstrated a low level of knowledge about key aspects of nasal irrigation, including the solution concentration, temperature, shelf life, target populations, clinical indications, and differences between irrigation devices. Patient age, educational level, and reading frequency significantly influence patient knowledge, attitudes, practice, and information acquisition related to nasal irrigation.

These findings highlight the need for more comprehensive, accessible, and diverse educational initiatives and clinical guidance to improve patients' understanding of nasal irrigation. By enhancing science popularization efforts, patients with nasal inflammatory diseases can achieve better treatment outcomes and optimize the therapeutic benefits of nasal irrigation.

## Data Availability

The raw data supporting the conclusions of this article will be made available by the authors, without undue reservation.
