# Supplementary material for: The knowledge, attitudes and practice of nasal irrigation among patients with rhinosinusitis: a cross-sectional study
Source: Front Allergy. 2026 Feb 6;6:1741401. doi: 10.3389/falgy.2025.1741401 (PMC12920464; doi:10.3389/falgy.2025.1741401)
Supplement: Supplementary file 1 [file Table1.docx]

**S1 Appendix**

**The questionnaire of the** **knowledge, attitudes and practice of nasal irrigation**

**General information**

1 Your age: ____ years

2 Your gender:

A. male

B. female

3 Your occupation:

A. farmer

B. worker

C. freelancer

D. professional/technical personnel

E. civil servant

F. student

G. without jobs

4 Your education level:

A. elementary school or below

B. middle school

C. college or above

5 Do you have a habit of reading?

A. yes, frequently

B. yes, occasionally

C. no

6 Your reading preference is:

A. story or fiction

B. news

C. popular science readings

D. professional readings

E. others

**Knowledge of nasal irrigation**

1. Whether tap water or purified water can be used?
2. yes

B. no

2 Normal saline must be used.

1. yes
2. no

3 Hypertonic saline can be used.

1. yes
2. no

4 Hypotonic saline can be used.

1. yes
2. no

5 Table salt mixed with water can be used for rinsing.

1. yes
2. no

6 Medications can be added at will.

1. yes
2. no

7 Nasal irrigation solution is preferably at room temperature.

1. yes
2. no

8 Nasal irrigation solution is preferably at body temperature.

1. yes
2. no

9 Nasal irrigation should be used within 24 hours.

1. yes
2. no

10 Electric or manual nasal irrigators have the same efficacy.

1. yes
2. no

11 Nasal spray and nasal irrigation are the same.

1. yes
2. no

12 When performing nasal irrigation, it is necessary to lean the upper body forward and lower the head.

1. yes
2. no

13 Nasal irrigation can only be conducted once daily.

1. yes
2. no

14 The frequency of nasal irrigation can be adjusted.

1. yes
2. no

15 The course of nasal irrigation is not fixed.

1. yes
2. no

16 Nasal irrigation can be used in children.

1. yes
2. no

17 Women in pregnancy can use nasal irrigation.

1. yes
2. no

18 Nasal irrigation can be used when nasal bleeding.

1. yes
2. no

**Attitudes toward nasal irrigation**

1 Nasal irrigation is safe.

1. definitely safe
2. safe
3. neutral
4. unsafe
5. definitely unsafe

2 Nasal irrigation can treat rhinosinusitis.

1. yes
2. no
3. uncertain

3 Nasal irrigation is important for rhinosinusitis treatment.

1. very important
2. important
3. neutral
4. unimportant
5. definitely unimportant
6. Nasal irrigation can help you relieve from rhinosinusitis.
7. yes
8. no
9. uncertain

**Practice of nasal irrigation**

1 Have you ever used nasal irrigation?

1. yes
2. no
3. Are you using nasal irrigation now?
4. yes
5. no

3 Nasal irrigation can be conducted by yourself.

1. yes
2. no

4 Nasal irrigation can alleviate your discomfort.

1. yes
2. no
3. uncertain
4. Nasal irrigation makes you uncomfortable.
5. yes
6. no
7. You can tolerate nasal irrigation.
8. yes
9. no
10. What would you do if nasal irrigation made you uncomfortable?
11. stop irrigation
12. continue irrigation
13. delay irrigation
14. uncertain
15. You would recommend nasal irrigation to others with nasal discomfort.
16. yes
17. no
18. uncertain

**Sources of nasal irrigation information and equipment**

1. Where did you know about nasal irrigation?
2. hospital
3. internet
4. popular science books/newspapers
5. acquaintances
6. Where did you learn about the knowledge of nasal irrigation?
7. hospital
8. internet
9. popular science books/newspapers
10. acquaintances
11. Where did you buy your nasal irrigation device?
12. hospital
13. internet
14. pharmacy
15. If you have problems about nasal irrigation, whom do you like to seek help from?
16. doctors or nurses
17. internet
18. manuals/professional books/newspapers
19. people have irrigated

**S1 Table**

**Factors influencing patients’ knowledge about nasal irrigation**

|  | count of correct answers  (M(IQR)) | B | P |
| --- | --- | --- | --- |
| **Age** |  | 0.235 | 0.132 |
| ˂50 years | 13 (9, 14) |  |  |
| ≥50 years | 9 (6, 11) |  |  |
| **Sex** |  | -0.029 | 0.839 |
| Male | 11 (7, 13) |  |  |
| Female | 10 (8, 13) |  |  |
| **educational attainment** |  |  |  |
| elementary school or below(reference) | 8 (6, 11) |  |  |
| middle school | 11 (7, 13) | 0.197 | 0.335 |
| college or above | 13 (9, 14) | 0.275 | 0.240 |
| **reading frequency** |  |  |  |
| frequently(reference) | 10 (8, 13) |  |  |
| occasionally | 12 (8, 13) | 0.104 | 0.586 |
| none | 10 (6, 13) | 0.122 | 0.594 |

M: median

IQR: interquartile ranges

B: regression coefficient

**S2 Table**

**Factors influencing the knowledge acquisition of nasal irrigation**

|  | **age**  (<50years/  ≥50 years) | | **sex**  (male/  female) | | **educational level**  (elementary school or below/ middle school/ college or above) | | | **reading frequency**  (frequently/  occasionally/  none) | |
| --- | --- | --- | --- | --- | --- | --- | --- | --- | --- |
|  | χ² | P | χ² | P | χ² | P | χ² | | P |
| **Item 1**  **Whether tap water or purified water can be used.** | 0.229 | 0.892 | 2.295 | 0.317 | 4.208 | 0.379 | 1.663 | | 0.797 |
| **Item 2**  **Normal saline must be used.** | 1.491 | 0.475 | 3.443 | 0.179 | 2.628 | 0.622 | 3.987 | | 0.408 |
| **Item 3**  **Hypertonic saline can be used.** | 4.593 | 0.101 | 3.676 | 0.159 | 1.867 | 0.760 | 3.563 | | 0.468 |
|  |  | |  | |  |  |  |  |  |
|  |  | |  | |  |  |  |  |  |
| **Item 4**  **Hypotonic saline can be used.** | 0.968 | 0.616 | 1.132 | 0.568 | 4.614 | 0.329 | 2.206 | | 0.698 |
| **Item 5**  **Table salt mixed with water can be used for rinsing.** | 2.242 | 0.326 | 1.015 | 0.602 | 4.006 | 0.405 | 2.931 | | 0.569 |
| **Item 6**  **Medications can be added at will.** | 9.211 | **0.010** | 0.244 | 0.885 | 0.748 | 0.945 | 2.196 | | 0.700 |
| **Item 7**  **Nasal irrigation solution is preferably at room temperature.** | 2.638 | 0.267 | 0.923 | 0.630 | 6.859 | 0.144 | 2.967 | | 0.563 |
| **Item 8**  **Nasal irrigation solution is preferably at body temperature.** | 7.416 | **0.025** | 0.883 | 0.643 | 0.875 | 0.928 | 0.167 | | 0.997 |
| **Item 9**  **Nasal irrigation should be used within 24 hours.** | 4.341 | 0.114 | 0.087 | 0.957 | 6.928 | 0.140 | 3.227 | | 0.521 |
| **Item 10**  **Electric or manual nasal irrigators have the same efficacy.** | 7.642 | **0.022** | 0.941 | 0.625 | 3.602 | 0.463 | 2.824 | | 0.588 |
| **Item 11**  **Nasal spray and nasal irrigation are the same.** | 6.925 | **0.031** | 0.291 | 0.865 | 2.828 | 0.587 | 0.990 | | 0.911 |
| **Item 12**  **Posture of nasal irrigation** | 5.021 | 0.081 | 0.932 | 0.627 | 0.958 | 0.916 | 4.741 | | 0.315 |
| **Item13**  **Nasal irrigation can only be conducted once daily.** | 13.180 | **0.001** | 2.181 | 0.336 | 1.586 | 0.811 | 13.040 | | **0.011** |
| **Item 14**  **The frequency of nasal irrigation can be adjusted.** | 6.809 | **0.033** | 1.851 | 0.396 | 3.832 | 0.429 | 6.303 | | 0.178 |
|  |  |  |  |  |  |  |  | | |
|  |  |  |  |  |  |  |  | | |
| **Item 15**  **The course of nasal irrigation is not fixed.** | 2.115 | 0.347 | 2.327 | 0.312 | 1.691 | 0.792 | 3.522 | | 0.475 |
| **Item 16**  **Nasal irrigation can be used in children.** | 16.245 | **<0.001** | 0.882 | 0.643 | 6.319 | 0.177 | 4.646 | | 0.326 |
| **Item 17**  **Women in pregnancy can use nasal irrigation.** | 2.818 | 0.244 | 1.114 | 0.573 | 5.689 | 0.224 | 2.736 | | 0.603 |
| **Item 18**  **Nasal irrigation can be used when nasal bleeding.** | 4.149 | 0.126 | 1.236 | 0.539 | 5.170 | 0.270 | 9.762 | | **0.045** |

**S3 Table**

**Factors influencing the attitudes of nasal irrigation**

|  | **age**  (<50years/  ≥50 years) | | **sex**  (male/  female) | | **educational level**  (elementary school or below/ middle school/ college or above) | | | **reading frequency**  (frequently/  occasionally/  none) | |
| --- | --- | --- | --- | --- | --- | --- | --- | --- | --- |
|  | χ² | P | χ² | P | χ² | P | χ² | | P |
| **Item 1**  **Nasal irrigation is safe.** | 3.380 | 0.337 | 8.035 | 0.045 | 11.319 | 0.079 | 7.641 | | 0.266 |
| **Item 2**  **Nasal irrigation can treat rhinosinusitis.** | 2.518 | 0.284 | 0.167 | 0.920 | 8.953 | 0.062 | 3.749 | | 0.441 |
| **Item 3**  **Nasal irrigation is important for rhinosinusitis treatment.** | 4.851 | 0.303 | 6.067 | 0.194 | 4.762 | 0.783 | 3.710 | | 0.882 |
|  |  | |  | |  |  |  |  |  |
|  |  | |  | |  |  |  |  |  |
| **Item 4**  **Nasal irrigation can help you relieve from rhinosinusitis.** | 0.769 | 0.681 | 1.002 | 0.606 | 2.460 | 0.652 | 3.274 | | 0.513 |
